# Methionine synthase A2756G polymorphism influences pediatric acute lymphoblastic leukemia risk: a meta-analysis

**DOI:** 10.1042/BSR20181770

**Published:** 2019-01-15

**Authors:** Li-Min Ma, Hai-Ping Yang, Xue-Wen Yang, Lin-Hai Ruan

**Affiliations:** Department of Hematology, The First Affiliated Hospital, and College of Clinical Medicine of Henan University of Science and Technology, Luoyang 471023, Henan Province, China

**Keywords:** leukaemia, methionine synthase, meta analysis, single nucleotide polymorphisms, susceptibility

## Abstract

Plenty of studies have investigated the effect of methionine synthase (*MTR*) A2756G polymorphism on risk of developing pediatric acute lymphoblastic leukemia (ALL), but the available results were inconsistent. Therefore, a meta-analysis was conducted to derive a more precise estimation of the association between *MTR* A2756G polymorphism and genetic susceptibility to pediatric ALL. The PubMed, Embase, Google Scholar, Web of Science, ScienceDirect, Wanfang Databases and China National Knowledge Infrastructure were systematically searched to identify all the previous published studies exploring the relationship between *MTR* A2756G polymorphism and pediatric ALL risk. Odds ratios (ORs) and 95% confidence intervals (CIs) were applied to evaluate the strength of association. Sensitivity analysis and publication bias were also systematically assessed. This meta-analysis finally included ten available studies with 3224 ALL cases and 4077 matched controls. The results showed that there was significant association between *MTR* A2756G polymorphism and risk of pediatric ALL in overall population (AG vs. AA: OR = 1.13, 95%CI = 1.02–1.26, *P* = 0.02; AG+GG vs. AA: OR = 1.13, 95%CI = 1.02–1.25, *P* = 0.01; G allele vs. A allele: OR = 1.10, 95%CI = 1.01–1.20, *P* = 0.03). In the stratification analyses by ethnicity, quality score and control source, significant association was found in Caucasians, population-based designed studies and studies assigned as high quality. In conclusion, this meta-analysis suggests that *MTR* A2756G polymorphism may influence the development risk of pediatric ALL in Caucasians. Future large scale and well-designed studies are required to validate our findings.

## Introduction

Acute lymphoblastic leukemia (ALL) is the most common childhood cancer, which accounts for 30% of all malignancy diagnosed in children and 80% of pediatric leukemia [1]. However, the etiology and biological mechanisms underlying ALL development have yet to be elucidated [2–4]. As for many cancers, the interactions between susceptibility genes and environmental factors are likely to implicate in the development of ALL. Epidemiological studies suggest that the imbalance of folate metabolism may be involved in predisposition to carcinogenesis, which is based on its involvement in both DNA biosynthesis and DNA methylation [5]. The low availability of folate causes uracil misincorporation into DNA replication, which leads to double-strand breakage and chromosomal deletion [6,7]. Moreover, gene-specific hypermethylation and global DNA hypomethylation are two of the most frequently observed altered DNA methylation patterns in tumors [8,9]. Accumulating studies have reported that polymorphisms in genes encoding folate-metabolizing enzymes disturb the balance of folate metabolism and have been associated with an altered predisposition to cancer [10–12].

The methionine synthase (MTR) plays a crucial role in the folate metabolic network. It is a vitamin B_12_-dependent enzyme, which remethylates homocysteine to methionine and simultaneously generates tetrahydrofolate by removing methyl group from 5-methyltetrahydrofolate. MTR helps to maintain the levels of adequate intracellular folate and normal homocysteine and methionine concentrations, which are used for proper DNA methylation or other methylation processes [13]. The *MTR* gene is mapped on 1q43, and the extensively investigated A2756G polymorphism (rs1805087) leads to a change from aspartate to glycine at codon 919 (D919G), resulting in reduced enzyme activity [14]. It has been reported that this polymorphism can increase homocysteine levels through suppressing methionine metabolism and consequentially can lead to DNA hypomethylation and promote tumorigenesis [15,16]. Plenty of studies have found that *MTR* A2756G polymorphism has been linked to various cancer, such as prostate cancer, retinoblastoma and lymphoma [17–19]. A number of studies have attempted to explore the effect of *MTR* A2756G polymorphism on pediatric ALL risk, yet the reported results are inconsistent. The inconsistencies of results might be attributed to some variables in study of population like genetic backgrounds difference and relatively small size of sampling in single study. Therefore, a meta-analysis was conducted to derive a more precise estimation of the association between *MTR* A2756G polymorphism and genetic susceptibility to pediatric ALL.

## Methods

### Identification and eligibility of relevant studies

The present study was conducted in accordance with the Preferred Reporting Items for Systematic Reviews and Meta-Analyses guidelines. The PubMed, Embase, Google Scholar, Web of Science, ScienceDirect, Wanfang Databases and China National Knowledge Infrastructure were systematically searched to identify the published case–control studies on the relationship between *MTR* A2756G polymorphism and pediatric ALL risk with the following subject terms or keywords: ‘methionine synthase’ or ‘MTR’ or ‘MS’ or ‘5-methyltetrahydrofolate-homocysteine methyltransferase’, ‘polymorphism’ or ‘variation’ or ‘variant’ or ‘mutation’, ‘acute lymphoblastic leukemia’ or ‘leukemia’ or ‘ALL’, and ‘pediatric’ or ‘children’ or ‘childhood’. The latest web-based literature search was conducted on May 20, 2018 and no language restriction was applied. In addition, the reference lists in the primary studies and review articles were also examined manually to identify additional potentially relevant studies.

### Inclusion criteria

The following inclusion criteria were applied for selecting literature: (1) confirmed diagnosis for the pediatric ALL cases; (2) case–control study; (3) available genotypes distribution data for both patients and control populations; (4) genotypes distribution of the control group must be in consistent with Hardy–Weinberg equilibrium (HWE). The case reports, letters, commentary and review articles were excluded. If the same or overlapping patient population was reported by several articles, only the most recent or largest sample size was chose in this meta-analysis.

### Quality assessment

The quality assessment of included studies was preformed independently by two authors according to the Newcastle-Ottawa Scale (NOS). Discrepancies were adjudicated by the third investigator until consensus was achieved. The NOS is a tool used for assessing the quality of non-randomized studies included in a systematic review and meta-analysis [20]. Using the tool, each study is judged on eight items, categorized into three groups: the study group selection, the comparability of the groups, and the ascertainment of exposure. Stars are awarded such that the highest quality studies are awarded up to nine stars. In this meta-analysis, studies with more than six stars were identified as high quality.

### Data collection

From each of the included articles, the following data were collected independently by two authors: the name of first author, year of publication, country and ethnicity of participants, source of controls, total number of cases and controls, genotyping methods, genotyping data of the *MTR* A2756G polymorphism in cases and controls. Any disagreement was resolved by re-evaluation of the originally included studies.

### Statistical analysis

For the controls of each study, the *χ^2^*-test was adopted to check HWE of genotypes distribution frequencies, with *P*<0.05 indicating deviation from HWE. The strength of association between *MTR* A2756G polymorphism and pediatric ALL risk was assessed by calculating pooled ORs and corresponding 95% CIs under the allele model (G allele vs. A allele), heterozygote model (AG vs. AA), homozygote model (GG vs. AA), recessive model (GG vs. AA+AG) and dominant model (AG+GG vs. AA), respectively. The significance of the overall ORs was determined by the *Z*-test. The *χ^2^*-test based *Q*-test was performed to estimate the heterogeneity across the eligible studies, and the heterogeneity was further quantified with *I*^2^-test. When *P*>0.05, showing that the effects were assumed to be homogeneous, the fixed-effects model (Mantel–Haenszel method) was selected to calculate the ORs, alternatively, the random-effects model (DerSimonian–Laird method) was used [21]. Stratification analyses were performed by ethnicity (Asian and Caucasian), control source (hospital-based and population-based) and NOS score (low quality and high quality). Sensitivity analysis was conducted by excluding one study each time and recalculating the ORs with corresponding 95%CIs to assess the stability of combined results. The qualitative funnel plot was employed to assess publication bias by calculating the standard error of log(OR) of each study plotted against its log(OR), and the funnel plot asymmetry was further assessed using quantitative Egger’s test [22]. All the statistical tests were done with RevMan v5.3 (The Cochrane Collaboration, Oxford, U.K.) and STATA v12.0 (Stata Corporation, College Station, TX). All *P* values were two-sided, and *P*<0.05 was considered statistically significant.

## Results

### Characteristics of included studies

**Figure 1:**
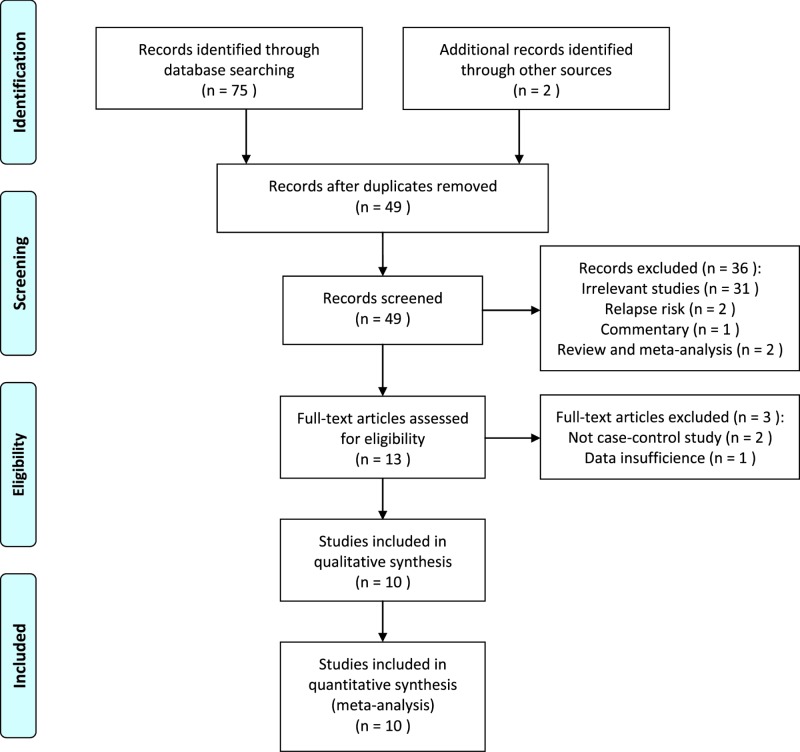
Flow diagram of study selection process

The flow diagram of literature selection was presented in Figure 1. After duplicates removed, 49 relevant articles were identified based on an extensive search. After glancing the titles and abstracts, 36 irrelevant studies and reviews were excluded and three full-text articles were excluded during the further assessment. Finally, a total of ten case–control studies met our inclusion criteria, including 3224 ALL cases and 4077 matched controls [23–32]. Table 1 presented the main characteristics of eligible studies. Of these included studies, there were nine studies carried out among Caucasians [23–31], and one study among Asian descents [32]. When classified by the source of controls, one study was hospital-based [26] and nine were population-based designed [23–25,27–32]. Three studies were divided into low quality and seven were assigned as high quality. The genotypes distribution frequencies among the controls were in agreement with HWE for all included studies. The genotyping data of *MTR* A2756G polymorphism in cases and controls from the individual studies were shown in Table 2.

**Table 1:** Main characteristics of studies included in the meta-analysis

Reference	Year	Country	Ethnicity	Control source	Genotyping methods	Quality score
de Jonge et al. [23]	2009	Netherlands	Caucasian	PB	PCR-RFLP	5
Gast et al. [24]	2007	Germany	Caucasian	PB	Allelic discrimination	8
Kamel et al. [25]	2007	Egypt	Caucasian	PB	PCR-RFLP	6
Lautner-Csorba et al. [26]	2013	Hungary	Caucasian	HB	MassARRAY	7
Lightfoot et al. [27]	2010	U.K.	Caucasian	PB	TaqMan Assay	9
Metayer et al. [28]	2011	U.S.A.	Caucasian	PB	GoldenGate Assay	8
Milne et al. [29]	2015	Australia	Caucasian	PB	PCR-RFLP	9
Petra et al. [30]	2007	Slovenia	Caucasian	PB	PCR-RFLP	5
Rahimi et al. [31]	2012	Iran	Caucasian	PB	PCR-RFLP	7
Nikbakht et al. [32]	2012	India	Asian	PB	PCR-RFLP	9

Abbreviations: HB, hospital-based; PB, population-based; PCR, polymerase chain reaction; RFLP, restriction fragment length polymorphism.

**Table 2:** Genotypes distribution of *MTR* A2756G polymorphism in cases and controls

Reference	Sample size		Case group					Control group					
	Case	Control	AA	AG	GG	A	G	AA	AG	GG	A	G	*P*_HWE_
de Jonge et al. [23]	245	489	162	74	9	398	92	340	137	12	817	161	0.68
Gast et al. [24]	446	547	280	153	13	713	179	375	151	21	901	193	0.24
Kamel et al. [25]	87	306	55	29	3	139	35	194	97	15	485	127	0.53
Lautner-Csorba et al. [26]	543	529	344	175	24	863	223	341	163	25	845	213	0.34
Lightfoot et al. [27]	870	759	531	288	51	1350	390	510	223	26	1243	275	0.79
Metayer et al. [28]	376	447	237	123	16	597	155	292	137	18	721	173	0.70
Milne et al. [29]	391	514	251	130	10	632	150	337	158	19	832	196	0.93
Petra et al. [30]	68	258	51	16	1	118	18	161	82	15	404	112	0.30
Rahimi et al. [31]	73	128	42	26	5	110	36	75	47	6	197	59	0.69
Nikbakht et al. [32]	125	100	74	44	7	192	58	58	35	7	151	49	0.59

Abbreviations: HWE, Hardy–Weinberg equilibrium; *MTR*, methionine synthase.

### Quantitative data synthesis

**Figure 2:**
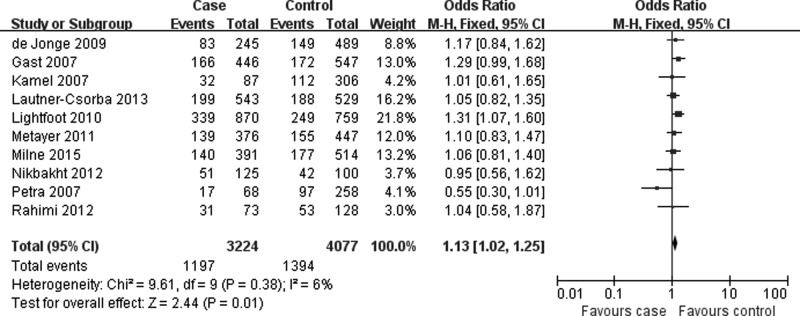
Forest plot describing the association of *MTR* A2756G polymorphism and pediatric ALL risk (AG+GG vs. AA)

**Figure 3:**
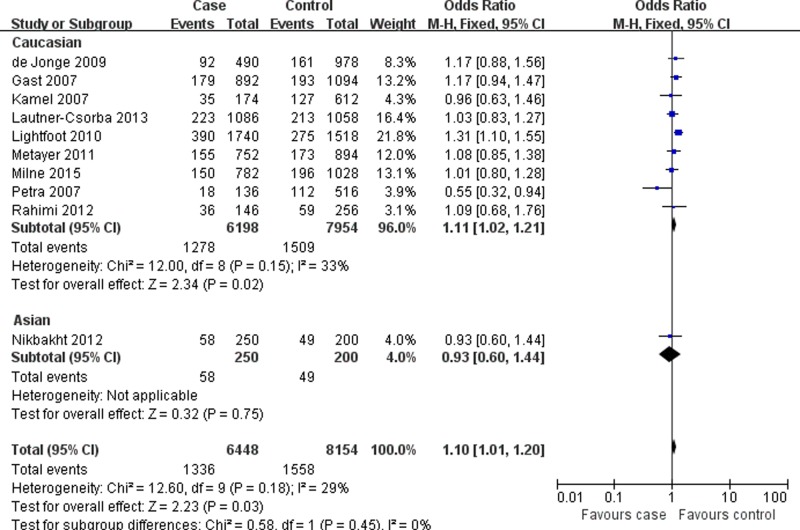
Forest plot describing the association of *MTR* A2756G polymorphism and pediatric ALL risk (G allele vs. A allele; stratified by ethnicity)

**Figure 4:**
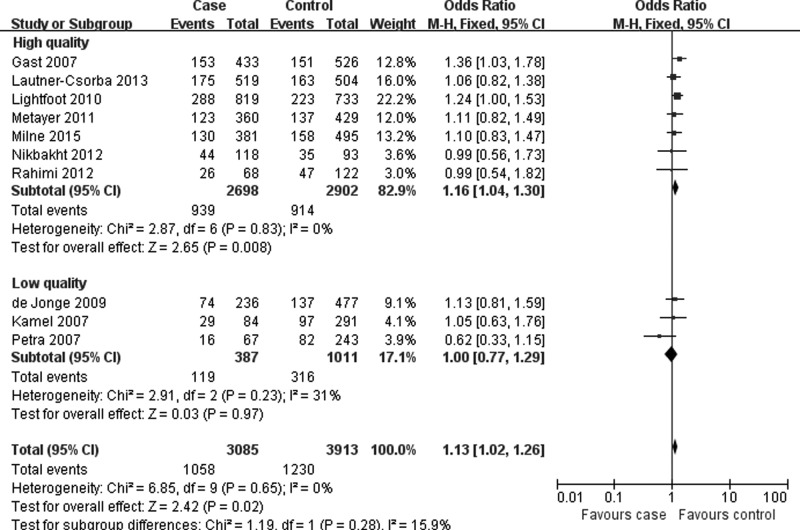
Forest plot describing the association of *MTR* A2756G polymorphism and pediatric ALL risk (AG vs. AA; stratified by quality score)

Table 3 listed the main results of quantitative synthesis. When all eligible studies were pooled together, the results found that there was statistically significant association between *MTR* A2756G polymorphism and risk of pediatric ALL under three genetic models (AG vs. AA: OR = 1.13, 95%CI = 1.02–1.26, *P* = 0.02; AG+GG vs. AA: OR = 1.13, 95%CI = 1.02–1.25, *P* = 0.01; G allele vs. A allele: OR = 1.10, 95%CI = 1.01–1.20, *P* = 0.03) (Figure 2 and Table 3). In the stratification analyses according to ethnicity, control source and quality score, significant association was also found in Caucasians, population-based designed studies, and studies assigned as high quality (Figures 3 and 4, Table 3).

**Table 3:** Results of meta-analysis for *MTR* A2756G polymorphism with pediatric ALL risk

Variables	No.	Sample size		AG vs. AA			GG vs. AA			GG vs. AA+AG			AG+GG vs. AA			G Allele vs. A Allele		
		Case	Control	OR (95% CI)	*P*	*P*_h_*	OR (95% CI)	*P*	*P*_h_*	OR (95% CI)	*P*	*P*_h_*	OR (95% CI)	*P*	*P*_h_*	OR (95% CI)	*P*	*P*_h_*
Overall	10	3224	4077	1.13 (1.02–1.26)	0.02	0.65	1.10 (0.86–1.39)	0.45	0.28	1.05 (0.83–1.34)	0.66	0.33	1.13 (1.02–1.25)	0.01	0.38	1.10 (1.01–1.20)	0.03	0.18
Ethnicity																		
Caucasian	9	3099	3977	1.14 (1.03–1.27)	0.01	0.58	1.11 (0.87–1.42)	0.39	0.23	1.07 (0.84–1.37)	0.58	0.27	1.14 (1.03–1.26)	0.01	0.33	1.11 (1.02–1.21)	0.02	0.15
Asian	1	125	100	0.99 (0.56–1.73)	0.96		0.78 (0.26–2.36)	0.66		0.79 (0.27–2.33)	0.67		0.95 (0.56–1.62)	0.86		0.93 (0.60–1.44)	0.75	
Control source																		
PB	9	2681	3548	1.15 (1.03–1.28)	0.02	0.58	1.13 (0.87–1.47)	0.37	0.23	1.08 (0.83–1.41)	0.55	0.27	1.15 (1.03–1.28)	0.01	0.33	1.11 (1.02–1.22)	0.02	0.15
HB	1	543	529	1.06 (0.82–1.38)	0.64		0.95 (0.53–1.70)	0.87		0.93 (0.53–1.65)	0.81		1.05 (0.82–1.35)	0.71		1.03 (0.83–1.27)	0.82	
Quality																		
High	7	2824	3024	1.16 (1.04–1.30)	0.008	0.83	1.14 (0.88–1.48)	0.32	0.29	1.09 (0.84–1.41)	0.52	0.31	1.16 (1.04–1.29)	0.006	0.71	1.12 (1.03–1.23)	0.01	0.49
Low	3	400	1053	1.00 (0.77–1.29)	0.97	0.23	0.86 (0.45–1.64)	0.65	0.16	0.87 (0.45–1.66)	0.67	0.20	0.98 (0.77–1.26)	0.89	0.10	0.90 (0.60–1.35)	0.60	0.05

* *P*_h_ value used to evaluate the heterogeneity between included studies. ALL, acute lymphoblastic leukemia; CI, confidence interval; HB, hospital-based; *MTR*, methionine synthase; OR, odds ratio; PB, population-based.

### Heterogeneity and sensitivity analysis

No significant heterogeneity was detected across the eligible studies under all five genetic models for *MTR* A2756G polymorphism, so the fixed-effects model based Mantel–Haenszel method was selected for the combined analysis (Table 3). Sensitivity analysis, in which the pooled ORs were recalculated after sequential omission of individual studies, revealed that the combined results remained virtually unchanged, suggesting the robustness of quantitative synthesis results.

### Publication bias

**Figure 5:**
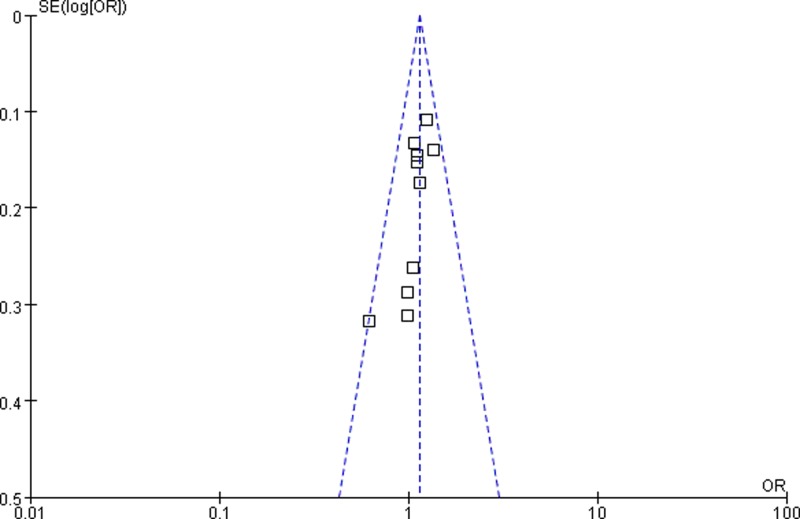
Funnel plot assessing publication bias in heterozygote model (AG vs. AA)

**Figure 6:**
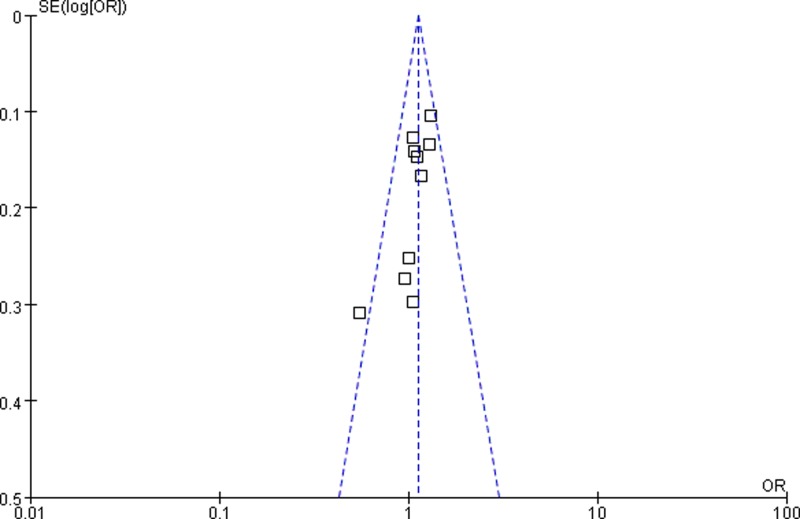
Funnel plot assessing publication bias in dominant model (AG+GG vs. AA)

The shapes of funnel plots appeared symmetrical, suggesting that there was no obvious publication bias (Figures 5 and 6). In addition, the results of Egger’s test also indicated a lack of publication bias of the current meta-analysis.

## Discussion

*MTR* gene encodes a vitamin B_12_-dependent enzyme, which catalyzes the remethylation of homocysteine to methionine, the precursor to S-adenosylmethionine, which acts as the universal methyl group donor [33]. The MTR reaction also releases tetrahydrofolate, which is remethylated to 5,10-methylene tetrahydrofolate for further participating in nucleotide synthesis. It is reported that *MTR* A2756G polymorphism can convert the codon for aspartate to glycine, resulting in a lower enzyme activity followed by homocysteine elevation and DNA hypomethylation [14,15]. In addition, the G-variant could enhance the flux of one-carbon moieties available for DNA methylation [13]. Therefore, *MTR* A2756G polymorphism might lead to alterations in DNA biosynthesis and methylation pattern, and contribute to the genetic susceptibility to cancer including leukemia, as hypermethylation is important in acute leukemia [34,35].

Numerous investigations have examined the association of *MTR* A2756G polymorphism with pediatric ALL susceptibility, yet have generated conflicting results. Petra et al. [30] found that the presence of at least one polymorphic *MTR* 2756 G allele showed some, but insignificant, tendency to reduce the risk for pediatric ALL. However, a dose–response relationship between the number of copies of the *MTR* 2756 G allele and increased risk of pediatric ALL was observed in the study by Lightfoot et al. [27]. Specifically, heterozygosity for the variant allele (AG) was associated with a 1.24-fold increased risk of ALL (95%CI = 1.00–1.53, *P* = 0.05), and homozygosity (GG) with a 1.88-fold increased risk of ALL (95%CI = 1.16–3.07, *P* = 0.01). de Jonge et al. [23] found no statistical differences in genotype distribution for *MTR* A2756G polymorphism between children ALL and the controls. To elucidate this inconsistency, a meta-analysis was conducted to derive a more precise estimation of the association.

In the present study, the combined results found that there was significant association between *MTR* A2756G polymorphism and risk of pediatric ALL in overall comparison. Individuals with the *MTR* 2756 G allele had increased risk of developing pediatric ALL compared to those with the A allele. Moreover, individuals with the AG genotype or the AG+GG genotype had raised risk of pediatric ALL compared to those with the AA genotype. Significant association was also found in Caucasians, population-based designed studies, and studies assigned as high quality. Our results were in accordance with the conclusion reported by Xia et al. [36], which showed *MTR* 2756 A allele was associated with a decreased risk of pediatric ALL compared with the G allele. In present meta-analysis, more web-based databases including English and non-English databases were systematically searched to minimize the selection bias and the potential risk of missing eligible literature [37]. Since our analysis included several new studies and included 3224 cases and 4077 controls, allowing for sufficient statistical power and more precise estimation, our conclusion is more credible.

When interpreting the results, some limitations of our meta-analysis should be considered. First, our results were based on unadjusted estimates, which may cause confounding bias. A more precise analysis could be performed if all raw data were available, which would allow for the adjustment by other confounders including sex, age, lifestyles and other potential factors. Second, the quantitative synthesis of some subgroups may have no sufficient testing power to accurately assess the real association, for instance, only one study was conducted among Asians. In addition, the gene–environment interactions which may modify genetic susceptibility to cancer were not taken into account in the present study due to the limited data. Last but not least, we also did not consider other genes in folate metabolic network that might be associated with the risk of pediatric ALL. The etiological mechanism of ALL is very complicated, in which gene–gene, and gene–environment interactions are involved [4,38]. Several case–control studies have reported that *MTR* 2756AG individuals who were *SHMT1* 1420CT/TT had a 5.6-fold reduction in ALL risk [39]. In contrast, *MTR* 2756 G was a risk allele for ALL on itself but also in combination with the *MTHFR* 677 T allele in adults [38]. The possibility cannot be ruled out that the role of *MTR* A2756G polymorphism is somewhat diluted or concealed by other gene–gene interactions. Future studies combining other genes in folate metabolism with *MTR* are encouraged.

## Conclusion

In conclusion, this meta-analysis suggests that *MTR* A2756G polymorphism influences the genetic susceptibility to pediatric ALL, especially in Caucasians. However, large scale and well-designed studies are required to validate our findings, and the biochemical mechanism and function of *MTR* A2756G polymorphism should also be investigated in the future.

## Abbreviations

ALL: acute lymphoblastic leukemia
CI: confidence interval
HB: hospital-based
HWE: Hardy–Weinberg equilibrium
MTHFR: methylenetetrahydrofolate reductase
MTR: methionine synthase
NOS: Newcastle–Ottawa Scale
OR: odds ratio
PB: population-based
PCR: polymerase chain reaction
RFLP: restriction fragment length polymorphism
SHMT1: serine hydroxymethyltransferase 1
